# Ireland’s Mental Health Bill 2024: progress, problems and Procrustean perils

**DOI:** 10.1007/s11845-024-03806-2

**Published:** 2024-09-27

**Authors:** Brendan D. Kelly

**Affiliations:** Department of Psychiatry, Trinity College Dublin, Trinity Centre for Health Sciences, Tallaght University Hospital, Tallaght, Dublin 24, D24 NR0A Ireland

**Keywords:** Human rights, Ireland, Legislation, Mental health, Psychiatry, Treatment

## Abstract

**Background:**

Ireland’s Mental Health Bill 2024 proposes the most significant revision of mental health legislation since the Mental Health Act 2001.

**Aims:**

To explore the 2024 Bill and provide suggestions for the subsequent Act.

**Methods:**

Review of the 2024 Bill and related literature.

**Results:**

The 2024 Bill proposes useful new definitions (e.g., ‘mental disorder’, ‘treatment’) and provisions governing specific practices (e.g., ‘physical restraint’). Revision is needed to better provide care and protect rights: (a) proposed treatment criteria for involuntary admission should be retained, but ‘risk’ criteria deleted; (b) treatment provisions should ensure *mental health legislation* provides for timely, accountable treatment for all patients; (c) detailed provisions about the content of treatment plans do not belong in primary legislation, which is ill-suited to micro-managing individual care and (d) the Mental Health Commission should be incorporated into the Health Information and Quality Authority.

**Conclusions:**

The 2024 Bill proposes useful changes but requires revision, especially for involuntary patients who lack decision-making capacity and decline care, for whom the Assisted Decision-Making (Capacity) Act 2015 does not (and was not designed to) provide solutions. Relying on a convoluted combination of the 2015 Act, Circuit Court and High Court would be legally impossible, clinically impracticable and *de facto* denial of the rights of people with serious mental illness and their families. The final Act can accord with principles of the 2015 Act without relying on its provisions and should benefit patients and support staff in delivering mental health care that is essential and often life-saving.

## Introduction

The Mental Health Bill 2024 was approved for publication by the Government on 24 July 2024 and published on 31 July 2024. The subsequent Act will be the most significant revision of mental health legislation since the Mental Health Act 2001. The 2001 Act was fully commenced in 2006 and would be repealed by the new Bill. The purposes of the 2024 Bill are, *inter alia*:

- ‘To provide for the admission to, and discharge from, registered acute mental health centres, of adult persons in certain circumstances and, for the involuntary admission of adult persons who meet the criteria for involuntary admission to such centres; to provide for the criteria for, and review of, involuntary admission of adult persons to registered acute mental health centres; and, for those purposes, to provide for the establishment of mental health review boards
- To provide for the care and treatment of adult persons in registered acute mental health centres and to provide that treatment, other than in certain limited circumstances, shall not be given to adult persons without consent being given for such treatment; to regulate the application, in certain limited circumstances, of restrictive practices in respect of adult persons who are admitted to registered acute mental health centres
- To provide for applications to be made to court to provide for the entitlements of persons admitted to registered acute mental health centres’
- To provide for the continuation in being of the Mental Health Commission and the Inspector of Mental Health Services
- To provide for the establishment and maintenance of a register of acute mental health centres, a register of community mental health centres and a register of community mental health services for such centres and services registered under and in accordance with this Act
- To provide for the regulation of mental health services including registered acute mental health centres, registered community mental health centres and registered community mental health services; and
- To provide for the monitoring and enforcement of compliance with the provisions of this Act by the Mental Health Commission’ (Mental Health Bill 2024; Preamble).

This paper summarises key provisions in the 2024 Bill as they relate to adults with mental disorder and provides suggestions for revisions to the final Act.^1^ Certain suggestions are provided throughout the paper. The “Discussion” section then focuses on four areas which merit particular attention in the development of the final Act: (a) its proposed criteria for involuntary admission (Section 12); (b) its proposed provisions for treatment of involuntary patients (Chapter 3 of Part 3); (c) its proposed provisions governing particular formats of care plans (Sections 179 and 181) and (d) its proposal that the Mental Health Commission (which was created by the Mental Health Act 2001) should continue to exist as a stand-alone entity (Section 92).

At this point, it is important to note that the clinical and legislative contexts of the new Bill are very different to those of the 2001 Act.

First, the intervening decades since the Mental Health Act 2001 have confirmed that psychiatric treatments for severe mental illness are considerably more effective than previously imagined, making an emphasis on providing treatment and support for severe mental illness even more urgent today [1–3]. Recent evidence confirms, for example, that antipsychotic medication in schizophrenia is associated with not only reduced symptoms of psychosis, but also substantially diminished risk of early death [4]. In this context, it is very helpful that the 2024 Bill aims ‘to provide for the care and treatment of adult persons in registered acute mental health centres’ (Preamble). This treatment is essential and often life-saving.

Second, as part of an increased emphasis on human rights, the necessity for treatment without consent in certain circumstances is increasingly accepted across a wide range of stakeholders and areas of health and social care, along with enhanced recognition of the validity of the concept of ‘decision-making capacity’ to consent to treatment. To take just one example, Gergel and colleagues, in a study of UK service-users, found that ‘the endorsement by the majority of service user respondents of involuntary treatment on the basis of impaired decision-making abilities counters a widespread view, upheld by the UN Committee on the Rights of Persons with Disabilities, that psychiatric use of capacity assessment and involuntary treatment necessarily violate fundamental human rights’ [5] (p. 600). Again, it is helpful that the 2024 Bill seeks to address these matters and provide necessary care for people with mental disorder even when they lack decision-making capacity to consent to such treatment and support.

### ‘Preliminary and General’ matters and principles of the Mental Health Bill 2024

Part 1 of the 2024 Bill concerns Preliminary and General matters and specifies that the Minister for Health can set commencement dates for various provisions (Section 1(2)). Section 2 presents definitions, including the following:

- An ‘adult’ is ‘a person who is 18 years of age or older’, and a ‘child’ is ‘a person under the age of 18’
- A ‘relative’ is ‘a parent, grandparent, child, grandchild, sibling, aunt, uncle or first-cousin of the person by blood, adoption, marriage or civil partnership’;
- A ‘spouse’ is ‘(a) the husband or wife, as the case may be, of the person, (b) the civil partner of the person, or (c) the cohabitant of the person’
- A ‘civil partner’ is ‘a person in a civil partnership or legal relationship to which section 3 of the Civil Partnership and Certain Rights and Obligations of Cohabitants Act 2010 applies’
- A ‘cohabitant’ is ‘one of 2 adults (whether of the same or opposite sex) who live together as a couple in an intimate and committed relationship and who are not married to each other or civil partners of each other’

Several groups of staff members are defined in the Bill:

- A ‘mental healthcare professional’ is ‘(a) a consultant psychiatrist, (b) a registered nurse within the meaning of section 2 (1) of the Nurses and Midwives Act 2011, (c) a member of one or more of the following designated professions within the meaning of section 3 of the Health and Social Care Professionals Act 2005, namely: (i) social worker; (ii) occupational therapist; (iii) speech and language therapist; (iv) such other designated profession within the meaning of the said section 3 of the said Act as the Minister [for Health] considers appropriate and may prescribe by regulations under section 3 of that Act’
- A ‘consultant psychiatrist’ is ‘a registered medical practitioner who is registered in the Specialist Division under the medical specialty of “Psychiatry”’
- A ‘specified person’ is ‘a person who (a) is appropriately trained to carry out any restrictive practice, (b) applies a restrictive practice under the direct supervision of a consultant psychiatrist or a relevant health professional, and (c) is employed by or otherwise works in a registered acute mental health centre or a designated centre’
- A ‘relevant health professional’ is ‘(a) a registered medical practitioner, (b) a registered nurse or registered midwife within the meaning of the Nurses and Midwives Act 2011, or (c) a registrant within the meaning of section 3 of the Health and Social Care Professionals Act 2005 prescribed for the purposes of this Part, who is appropriately trained to order the application, initiate the application of, or apply, a restrictive practice’

In relation to assessment, the proposed legislation introduces a significant change to the definition and use of the term ‘mental disorder’ which, in the Mental Health Act 2001, comprised the criteria for involuntary admission, but is applied differently in the 2024 Bill:

- ‘Mental disorder’ is ‘any mental disorder, illness or disability, whether of a continuous or intermittent nature, which affects the person’s thinking, perception, emotion, mood or judgment and impairs the mental function of the person’;
- An ‘examination’, ‘in relation to a recommendation, an involuntary admission order or a renewal order of any person for any purpose under this Act, means a personal examination carried out by a registered medical practitioner or a consultant psychiatrist of the process and content of thought, the mood, perceptions and the behaviour of the person concerned in order to make a diagnosis or a preliminary diagnosis’
- ‘Capacity’ has the same meaning as it has in Section 2 of the Assisted Decision-Making (Capacity) Act 2015.

The Bill states that ‘treatment’ ‘includes the administration of physical, psychological and other remedies relating to the care and rehabilitation of the person under clinical supervision, intended for the purposes of ameliorating a mental disorder or other mental health difficulty, and includes any relevant ancillary treatment and tests required for the purposes of safeguarding the person’s life or ameliorating the person’s condition’. This is a welcome re-definition of treatment which appears to include such essential interventions as re-feeding in severe anorexia nervosa, mandatory blood testing with clozapine treatment and various other matters.

The 2024 Bill also states that the Minister for Health ‘may by regulations provide for any matter referred to in this Act as prescribed or to be prescribed’ (Section 3(1)); repeals the Mental Health Act 2001 and Mental Health (Amendment) Act 2018 (Section 6); outlines ‘transitional provisions’ (Section 5) and presents provisions relating to ‘service of documents’ (Section 4), ‘expenses’ (Section 7) and a ‘review of operation of Act’ to occur ‘not later than 10 years after the commencement of this section’ (Section 8(1)). The 2024 Bill also outlines ‘guiding principles to apply in respect of adults’ (Section 9; Box 1).

**Box 1:** ‘Guiding principles to apply in respect of adults’ in Ireland’s Mental Health Bill 2024 (Section 9)

The Mental Health Bill 2024 outlines ‘guiding principles to apply in respect of adults’ which ‘shall apply in respect of the making of any decision relating to a voluntarily admitted person or an involuntarily admitted person (in this section referred to as an ‘applicable person’) in a registered acute mental health centre’ (Section 9(1)):
• ‘It shall be presumed that an applicable person has capacity to make decisions affecting himself or herself unless the contrary is shown in accordance with (a) *Part 3* [‘involuntary admission’], or (b) the provisions of the [Assisted Decision-Making Capacity Act 2015]’ (Section 9(2));
• ‘An applicable person shall not be considered as unable to make a decision affecting himself or herself unless all practicable steps have been taken, without success, to help him or her to do so, including by giving the applicable person concerned an opportunity, if he or she so wishes, to consult with a person or persons of his or her choosing prior to making such a decision’ (Section 9(3));
• ‘An applicable person shall not be considered as unable to make a decision merely by reason of making, having made, or being likely to make, an unwise decision’ (Section 9(4));
• ‘Where an applicable person lacks capacity in respect of the making of a decision then the provisions of the Act of 2015 shall apply in respect of the making of that decision except where provided for in *Chapter 3* of *Part 3*’ [‘Consent to treatment’] (Section 9(5));
• ‘Where it is proposed to make a decision in respect of an applicable person
(a) the applicable person shall be notified of the proposed decision in a form and language that may reasonably be understood by him or her,
(b) the applicable person shall be entitled to make representations in relation to the proposed decision,
(c) the applicable person shall be encouraged and facilitated to participate, or to improve his or her ability to participate, as fully as possible, in the decision, and
(d) all representations made by the applicable person to the person making the decision shall be taken into account before any decision is made’ (Section 9(6));
• In making a decision in relation to an applicable person, the person making the decision
(a) shall act at all times in good faith,
(b) may, with the consent of the applicable person concerned, consider the views of (i) any person engaged in caring for the applicable person, and (ii) any other mental healthcare professional,
(c) shall not seek to obtain information that is not reasonably required for the making of the decision,
(d) shall not use information for a purpose other than in relation to the proposed decision, and
(e) shall take all necessary steps to ensure that information (i) is kept secure from unauthorised access, use or disclosure, and (ii) is safely disposed of when he or she believes it is no longer required’ (Section 9(7)); and
• ‘A decision made in respect of an applicable person shall
(a) be made in a manner that minimises any restrictions of that person’s rights and freedoms,
(b) respect the right of the applicable person to dignity, bodily integrity, privacy and autonomy,
(c) be proportionate to the significance and urgency of the matter the subject of the decision,
(d) be limited in duration, in so far as is practicable, after taking into account the particular circumstances of the matter the subject of the decision,
(e) be made in a manner that promotes the highest attainable standard of mental health, subject to the availability of resources, and
(f) be made with due regard to the person’s will and preferences in relation to the decision’ (Section 9(8)).

## Involuntary admission in the Mental Health Bill 2024

The 2024 Bill outlines new ‘Criteria for involuntary admission to registered acute mental health centre’ (Section 12). These criteria differ substantially from those recommended in the *Report of the Expert Group on the Review of the Mental Health Act 2001* [6] (p. 22) and are outlined in Box 2. It is recommended that these criteria are substantially revised in the final Act; specific suggestions are provided in the “Discussion” section of this paper (below).

**Box 2:** ‘Criteria for involuntary admission to registered acute mental health centre’ in Ireland’s Mental Health Bill 2024 (Section 12(1))

‘A person may be involuntarily admitted to a registered acute mental health centre pursuant to an involuntary admission order and detained there if he or she fulfils each of the criteria (in this Act referred to as the “criteria for involuntary admission”) specified in either *paragraph (a)* or *(b)*, namely:
(a) the person has a mental disorder, the nature and degree of which is such that—
(i) the life of the person, or that of another person, is at risk, or the health of the person, or that of another person, is at risk of immediate and serious harm, and
(ii) if the first-mentioned person were to be admitted to and detained in a registered acute mental health centre (I) his or her admission and detention would be likely to reduce the risk he or she poses to himself or herself or others due to his or her mental disorder, (II) he or she would be likely to benefit from care and treatment that cannot be given to that person other than in a registered acute mental health centre, or (III) his or her admission and detention would be likely to benefit the condition of that person;
or
(b) the person has a mental disorder, the nature and degree of which is such that—
(i) he or she requires care and treatment immediately,
(ii) the care and treatment required to be given to the person cannot be given to that person other than in a registered acute mental health centre, and
(iii) the reception, detention and care and treatment of the person concerned in a registered acute mental health centre would be likely to benefit the condition of that person.’

The Bill adds that a person cannot be involuntarily admitted ‘by reason only of the fact that the person (a) has a mental disorder that does not fulfil the criteria for involuntary admission, (b) has an intellectual disability, (c) has a personality disorder, (d) is addicted to drugs or intoxicants, (e) may behave in such a manner or hold views that are contrary to, deviate from or transgress cultural, religious, social or traditional norms or customs of appropriate behaviour, or (f) requires to reside in a safe environment provided by a registered acute mental health centre’ (Section 12 (2)). The Mental Health Commission ‘shall prepare and issue a code of practice’ in this area (Section 12(3)).

### Applications for involuntary admission

The 2024 Bill suggests significant changes to applications for involuntary admission. There will be two kinds of applications: one made by an ‘authorised officer’ and one made by a ‘direct applicant’, which is a person other than an authorised officer. An authorised officer is ‘an officer of the Executive [i.e., ‘Health Service Executive’; HSE] who is of a prescribed rank or grade and who is authorised by the Director General’ of the HSE for this role (Section 2).

Section 13(1) states that, ‘where a person reasonably believes that another person (other than a child) has a mental disorder that fulfils the criteria for involuntary admission, the first-mentioned person […] may (a) request an authorised officer, or (b) request the Executive to nominate an authorised officer, to make an application […] for a recommendation for involuntary admission’. Various disqualifications apply, including the exclusion of ‘a member of An Garda Síochána [police] acting in the course of his or her duty’ (Section 13(2)(e)).

Following receipt of such a request, ‘the authorised officer shall, as soon as practicable […], assess whether to make an application to a registered medical practitioner for a recommendation for involuntary admission [and] shall (a) meet with, speak to and observe the person who is the subject of the request, (b) consult, where possible and appropriate, with that person’s spouse, relative or carer, as the case may be, and (c) take account of whether the care and treatment required to be given to the person can be given other than in a registered acute mental health centre, with a view to ensuring an application for a recommendation for involuntary admission is made only where necessary and appropriate’ (Section 14(1)). If the ‘authorised officer has reasonable grounds for believing that the person […] has a mental disorder which fulfils the criteria for involuntary admission, the authorised officer shall make an application’ (Section 14(3)) and state their reasons for making (Section 14(4)) or not making it (Section 14(6)). An ‘application shall be valid for 7 days’ (Section 14(9)) and various exclusion criteria apply (Section 14(11)).

The second kind of application is a ‘direct’ application by a ‘relevant person’. Section 15 of the Bill states that ‘a relevant person (in this Part referred to as a “direct applicant”) may make an application for a recommendation for involuntary admission in respect of a person directly to a registered medical practitioner […] where he or she believes that the person the subject of the application has a mental disorder that fulfils the criteria for involuntary admission’ (Section 15(1)). A ‘relevant person’ is ‘a person who (a) is a spouse or relative of the person, the subject of the application, (b) has a *bona fide* interest in the welfare of the person concerned, or (c) is a mental healthcare professional (other than a consultant psychiatrist)’ (Section 15(6)). Various disqualifications apply, including the exclusion of ‘a member of An Garda Síochána acting in the course of his or her duties’ (Section 15(2)(e)).

Section 15 (3) states that:

‘Prior to making a direct application for a recommendation for involuntary admission, a direct applicant shall, not more than 24 hours before the date of the making of the application—

- meet with, speak to, and observe the person, the subject of the application, and
- consider, to the best of his or her ability, whether there are reasonable grounds for believing that the person, the subject of the application, has a mental disorder which fulfils the criteria for involuntary admission.’

These provisions would represent a certain degree of change, although, while authorized officers are mentioned first, most persons who could apply for involuntary admissions under the 2001 Act appear to potentially qualify as a ‘relevant person’ in the 2024 Bill, with the notable exception of Gardaí, who can request that an application be made under Section 18 (below).

### Recommendations for involuntary admission

A ‘registered medical practitioner’ to whom an application is made ‘shall, within 24 hours of the receipt of the application carry out an examination of the person concerned to assess whether that person has a mental disorder which fulfils the criteria for involuntary admission’ (Section 16(1)). The doctor ‘shall inform the person […] of the purpose of the examination unless the provision of such information would, in the view of the registered medical practitioner concerned, seriously endanger the life or health of the person, […] or the life or health of another person or persons’ (Section 16(2)). If the doctor concludes ‘that the person has a mental disorder which fulfils the criteria for involuntary admission, [they] shall within 24 hours of the examination make a recommendation to the clinical director of a registered acute mental health centre (other than the Central Mental Hospital) that the person be involuntarily admitted’ there (Section 16(3)).

The Bill adds that that the examination can precede receipt of the application by a maximum of 24 hours, if necessary (Section 16(6)). A recommendation for involuntary admission shall be valid for a period of 7 days (Section 16(9)). Various disqualifications apply (Section 16(7)), and there are provisions for ‘disclosure of previous application for involuntary admission’ (Section 17).

These provisions are broadly similar to the 2001 Act, with the addition that a doctor’s recommendation can be based on an examination that precedes receipt of the application, thus removing the need for a doctor to re-examine a person after receiving an application if they have already examined the person in the preceding 24 hours. This will hopefully avoid repetitive, intrusive and potentially distressing re-examinations and is consistent with the *Report of the Expert Group on the Review of the Mental Health Act, 2001* which suggested ‘that the sequencing of whether the Authorised Officer or the Registered Medical Practitioner sees the patient first is not relevant once they are undertaken independently’ [6] (p. 37).

As regards transport, ‘the requester or direct applicant, as the case may be, shall arrange for the person the subject of the recommendation to be brought to the registered acute mental health centre specified in the recommendation as soon as practicable but not later than 12 hours’ after the ‘recommendation is made’ (Section 19(1)). If they are unable to do so, ‘the requester or direct applicant, as the case may be, or the registered medical practitioner […] shall request (a) the clinical director of the registered acute mental health centre specified in the recommendation, or (b) a consultant psychiatrist acting on that clinical director’s behalf, to arrange for the person concerned to be brought to the registered acute mental health centre’ (Section 19(2)). That person ‘shall arrange’ for this to happen (Section 19(3)), with Garda assistance if ‘such assistance is necessary to protect the health of the person concerned, or other persons from the threat of immediate and serious harm’ (Section 19(4)). Additional provisions cover ‘Bringing and bringing back of persons to registered acute mental health centre by a service provider’ (Section 20).

### Powers of Garda Síochána (police) in respect of involuntary admissions

Section 18 outlines the ‘powers of Garda Síochána in respect of involuntary admissions’:

‘Where a member of An Garda Síochána has reasonable grounds for believing that a person has a mental disorder that fulfils *paragraph (a)* of the criteria for involuntary admission [i.e., the ‘risk’ criteria only] the member may, either alone or with any other member or members of An Garda Síochána—

- take the person into custody and arrange for the matters specified in *subsections (3)* and *(4)* [application for involuntary admission] to be carried out as soon as practicable, but no later than 6 hours after the time that the person is taken into custody, and
- enter if needs be by force any dwelling or other premises or any place if he or she has reasonable grounds for believing that the person is to be found there’ (Section 18(1))

The 6-hour period ‘may be extended by one additional period of 6 hours under the authorisation of a Superintendent or Chief Superintendent’ if necessary (Section 18(2)).

During this time, the Garda ‘shall request (a) an authorised officer, or (b) the [HSE] to nominate an authorised officer, to make an application’ for involuntary admission (Section 18(3)). ‘Where, following the making of reasonable efforts by the [HSE], it has not been possible for an authorised officer to assess whether to make an application’, a Garda ‘shall request a relevant person’ to do so (Section 18(4)). Following such an application, a registered medical practitioner shall examine the person and make (or not make) a recommendation within the same time period (Section 18(6)), or else the person is released from custody (Section 18(7)).

If a recommendation is made, either the Gardaí ‘shall bring the person to the registered acute mental health centre’ or they ‘shall contact the clinical director of the registered acute mental health centre to arrange for the person to be brought to the registered acute mental health centre by its members of staff or a service provider as soon as practicable but not later than 6 hours after the time the recommendation is made’ (Section 18(8)).

### Emergency treatment before involuntary admission

After a recommendation for involuntary admission has been made, if the recommending doctor, clinical director, or psychiatrist ‘reasonably believes that the person […] requires emergency treatment, he or she may arrange for the person […] to be brought to a hospital for such emergency treatment as soon as practicable’ (Section 21(1)), after which involuntary admission can resume. ‘Emergency treatment’ is ‘any medical or dental treatment provided in a hospital other than a registered acute mental health centre which is urgent and immediately necessary to avoid significant harm, injury or death to the person’ (Section 21(6)). This treatment is not provided under the mental health legislation (Section 21(4)), i.e., the fact that the person is in the process of involuntary admission does not ‘affect any enactment or rule of law relating to consent to medical treatment’ (Section 21(5)).

### Involuntary admission order

When the ‘clinical director receives a recommendation for involuntary admission’, they ‘shall as soon as may be arrange for’ a consultant psychiatrist ‘to carry out an examination’ and ‘prior to, at the same time as or following’ such an examination, ‘a mental healthcare professional (other than a consultant psychiatrist) on the staff of the registered acute mental health centre who is or will be involved in the care and treatment of the person […] to carry out a psychosocial assessment’ (Section 22(1)). Following the ‘examination’, the consultant psychiatrist shall either make an ‘involuntary admission order’ or decide not to do so (Section 22(2)), within 24 hours of the person’s arrival (Section 22(3)).

A ‘psychosocial assessment’ is ‘a personal assessment carried out by a mental healthcare professional (other than a consultant psychiatrist) to assess (a) the psychological condition of the person, (b) the environmental and social factors that have contributed to his or her condition, (c) the ability of the person to care for himself or herself if he or she were to be not admitted to, or discharged from, as the case may be, a registered acute mental health centre, and (d) the supports that may be required and available to that person outside of the registered acute mental health centre’ (Section 11). The ‘conclusions from a psychosocial assessment […] shall be recorded in that person’s medical records’ (Section 22(4)).

An ‘involuntary admission order shall (a) authorise the reception, detention, care and treatment of the involuntarily admitted person concerned, and (b) [unless extended by the consultant psychiatrist or revoked by a mental health review board] remain in force for 21 days after the date of its making, and then expire’ (Section 23(1)). The order may be extended by periods of up to 3 months by a consultant psychiatrist (Sections 23(2–3)). They must have examined the patient within the previous 48 hours (Section 23(4)(a)) and ‘consult with a mental healthcare professional (other than a consultant psychiatrist) on the staff of the registered acute mental health centre who is or will be involved in the care and treatment of the involuntarily admitted person regarding the proposed renewal order’ (Section 23(4)(b)).

Additional provisions cover sending involuntary admission and renewal orders to the Mental Health Commission (Section 24) and the ‘provision of information to persons involuntarily admitted’ including (i) ‘a general description of the proposed care and treatment to be administered’ (Section 25(3)(b)); (ii) that they are ‘entitled to discuss discharge planning with a member of his or her multidisciplinary team’ (Section 25(3)(l)) and (iii) that they are ‘entitled to have a nominated person accompany him or her to meetings and to consult with him or her on decisions regarding the proposed care and treatment’ (Section 25(3)(m)).

## Reviews of involuntary admission

### Mental health review boards

Mental health tribunals are to be re-named ‘mental health review boards’. Each review board shall continue to comprise one consultant psychiatrist, one practising barrister or solicitor (with not less than 7 years’ experience) and one ‘member of a community review panel’ (Section 26). The Mental Health Commission shall create a ‘panel of independent consultant psychiatrists’ as is presently the case (Section 27). The powers of review boards are similar to those of tribunals, including notifying relevant parties ‘of the time and date for the review hearing’ (Section 28(6)).

The Mental Health Commission ‘shall publish on its website on a quarterly basis and in such form and manner as it considers appropriate, anonymised versions of every decision of a review board’ (Section 28(12)), although ‘where the Commission is of the view that, […] due to the existence of exceptional circumstances, the publication of a case […] would nonetheless identify the parties in relation to whom the decision relates, it may make a determination that the decision should not be published by the Commission’ (Section 28(13)). This is consistent with the *Report of the Expert Group on the Review of the Mental Health Act 2001* which recommended allowing ‘information in relation to decisions of Review Boards to be published in anonymised form which will ensure patient confidentiality. This will allow such decisions to be available for the Mental Health Commission and/or the public to view’ [6] (p. 49).

Additional proposals govern ‘provision of information to legal representatives’ (Section 29) and ‘referral’ of orders to review boards by the Mental Health Commission (Section 30). The review board will then, among other matters, ‘set the time and date for the hearing’ (Section 30(1)(b)), arrange examination by ‘an independent consultant psychiatrist’ (Section 30(1)(e)) and ‘request the clinical director of the registered acute mental health centre to arrange for a mental healthcare professional (other than a consultant psychiatrist) on the staff of the registered acute mental health centre who is or will be involved in the care and treatment of the person concerned to carry out a psychosocial assessment’ (Section 30(1)(d)). That report is to be prepared and submitted ‘not less than 3 working days prior to the hearing’ (Section 30(2)). Finally, ‘the responsible consultant psychiatrist may, but is not obliged to, submit a report to the Commission as soon as may be and in any event no later than 3 working days prior to the review board hearing’ (Section 30(6)).

One of the chief functions of mental health review boards is to review involuntary admission and renewal orders within 21 days, and either affirm or revoke the relevant order, using criteria similar to those of the 2001 Act (Section 31). The 21 day-period can be extended for two periods of up to 7 days each in certain circumstances (Section 31(4)).

### Appeal to the circuit court and high court

The 2024 Bill states that ‘an involuntarily admitted person may appeal to the Circuit Court against a decision of a review board […] on the grounds that (a) he or she does not meet the criteria for involuntary admission, or (b) the provisions of *sections 9* to *24* and *37*, where applicable, were not complied with, and the failure affected the substance of the order and caused an injustice and the failure to comply with the provisions concerned was such as to render the detention invalid’ (Section 32(1)). The Court can either affirm or revoke the order, as appropriate, with the burden of proof lying on the registered acute mental health centre (Section 32(4)). ‘No appeal shall lie against an order of the Circuit Court under this section other than an appeal on a point of law to the High Court’ (Section 32(16)).

## Change of status from voluntary to involuntary

If ‘a responsible consultant psychiatrist or another mental healthcare professional who is responsible for or involved in the care and treatment of the voluntarily admitted person is of the opinion that the voluntarily admitted person fulfils the criteria for involuntary admission’, they may detain that person ‘for a period not exceeding 24 hours’ (Section 37(1)). This would no longer be limited to doctors and nurses, and it would no longer be necessary for the person to express a desire to leave. The psychiatrist shall examine the person and ‘arrange for another mental healthcare professional (other than a consultant psychiatrist) on the staff of the registered acute mental health centre who is not involved in the care and treatment of the person […] to carry out a psychosocial assessment’ (Section 37(2)). This measure seems poorly formulated; psychiatrists do not have authority over other team members from different disciplines, and so cannot ensure that such a report is prepared. However, the change to enabling detention whether a person expresses a wish to leave or not is welcome. It will better protect the rights of those patients who are not detained, are severely unwell and require treatment, but are unable to consent.

If the responsible consultant psychiatrist concludes that the person ‘has a mental disorder which fulfils the criteria for involuntary admission, the consultant psychiatrist shall arrange for another consultant psychiatrist who is not on the staff of the registered acute mental health centre and who will not be involved in the care and treatment of the person […] to carry out a second examination’ (Section 37(5)). The second consultant psychiatrist shall also consult with the responsible consultant psychiatrist and consider the psychosocial assessment before deciding if the ‘person fulfils the criteria for involuntary admission’ (Section 37(6)).

## Information, transfers and leave for involuntary patients

Section 41 of the Bill addresses the provision of information to voluntary patients, stating that they are ‘entitled to have a nominated person […] accompany him or her to meetings and to consult with on decisions regarding the proposed care and treatment’ (Section 41(1)(h)). The information to which a ‘nominated person’ is entitled is outlined (Section 41(2)).

Additional provisions govern the transfer of an involuntary patient to another registered acute mental health centre at the request of the patient (Section 33), to another registered acute mental health centre (other than the Central Mental Hospital) by the clinical director (Section 34), to the Central Mental Hospital (which requires authorisation by a mental health review board) (Section 35) and ‘to hospital or other place in certain circumstances’ for ‘treatment and for his or her detention there for that purpose’ (Section 36)(1)).

A responsible consultant psychiatrist may grant leave to an involuntary patient for a period of up to 14 continuous days ‘subject to such conditions as the consultant psychiatrist considers appropriate and so specifies in writing’ (Section 38(1)). This can be extended for further periods of up to 14 days (Section 38(4)). The Mental Health Commission ‘shall publish a code of practice in relation to this section’ (Section 38(6)).

If an involuntary patient is absent without leave, the clinical director ‘shall arrange, for a member or members of the staff of the centre or other person or persons duly authorised in that behalf […] to bring the person back to the registered acute mental health centre’ (Section 39(1)), with the assistance of An Garda Síochána if needed (Sections 39(2) and 39(3)).

Provisions governing discharge of involuntary patients are similar to those in the 2001 Act (Section 40). If an involuntary patient is discharged before a mental health review board has reviewed the order, and if they request such a review:

‘[…] the review board shall review the detention of the person concerned and shall determine whether—

- (i) the provisions of *sections 9* to *24* and *37*, where applicable, were complied with, or (ii) if there was a failure to comply with any such provision, if the failure affected the substance of the order and caused an injustice, and
- the consultant psychiatrist who made the involuntary admission order or renewal order did so on the date the order was made on the basis that the person fulfilled the criteria for involuntary admission at the time of the making of the involuntary admission order or renewal order, as the case may be’ (Section 40(11)).

This changes the position from the 2001 Act by placing (b) clearly in the past tense, pertaining to time of involuntary admission rather than the day of the mental health review board hearing.

## Consent to treatment for involuntary patients

The 2024 Bill states that ‘a mental healthcare professional shall, before treating an involuntarily admitted person, obtain consent for the treatment concerned from the involuntarily admitted person, unless otherwise expressly provided for in this Act’ (Section 42). This is consistent with Section 57 of the 2001 Act, and capacity is assumed (Section 43(3)).

If ‘a responsible consultant psychiatrist reasonably considers that an involuntarily admitted person may lack capacity to consent to or refuse treatment, then he or she shall carry out a capacity assessment or arrange for another mental healthcare professional who is involved in the care and treatment of the person concerned to carry out the capacity assessment’ (Section 45(1)).^2^ If they conclude that the person lacks capacity, ‘the consultant psychiatrist shall arrange for a second capacity assessment of the person to be carried out by a second consultant psychiatrist or other mental healthcare professional’ (Section 45(2)) who is not ‘involved in the care and treatment of the person concerned’ (Section 45(3)).^3^

If the second assessment concludes that the patient ‘does not lack capacity, then that person shall be assessed as having capacity to consent to the proposed treatment’ (Section 45(5)). If both assessments indicate that the patient ‘lacks capacity to consent to or to refuse a proposed treatment, then that person shall be assessed as lacking capacity’ (Section 45(4)). In this circumstance, and in the absence of a decision-making representative or valid, relevant advance healthcare directive under the Assisted Decision-Making (Capacity) Act 2015, ‘an application shall be made by or on behalf of the responsible consultant psychiatrist to the Circuit Court under Part 5 of the Act of 2015 prior to any treatment being provided to the involuntarily admitted person’ (Section 46).

If the patient lacks capacity, ‘treatment may be administered to him or her if’:

- ‘in a case where there is a decision-making representative duly authorised by the Circuit Court to make decisions relevant to the person’s healthcare and treatment in accordance with the Act of 2015, the decision-making representative concerned consents to the treatment proposed, so long as it is not contrary to the known will and preferences of the person concerned,
- in a case where there is a valid and relevant advance healthcare directive in respect of the person which is relevant to the proposed treatment, a provision, or provisions, of the directive provides, or provide, for consent to the specific treatment proposed, or
- in a case where the Circuit Court has made a decision-making order under section 38 of the Act of 2015, the order of the Court provides for consent to the specific treatment proposed, so long as it is not contrary to the known will and preferences of the person concerned’ (Section 47(1))

If such arrangements are in process but not yet completed, treatment (other than electro-convulsive therapy)^4^ ‘may be given to the involuntarily admitted person concerned if such treatment is (i) immediately necessary for the protection of life of the person or that of another person, or (ii) necessary for protection from an immediate and serious threat to the health of the person, or that of other persons, and there is no alternative safe and effective treatment available’ (Section 47(2)).

A High Court application is required for treatment if the involuntary patient ‘(a) has capacity to make decisions about his or her treatment but refuses to consent to the treatment concerned’; (b) has a relevant support in place under the 2015 Act, but there is still no consent, or (c) the person lacks capacity ‘but the person’s will and preferences are known and those will and preferences are to refuse to consent to the treatment’ (Section 51(1)). In that circumstance, ‘an application may be made by or on behalf of the responsible consultant psychiatrist to the High Court specifying the proposed treatment and seeking an order to administer the treatment concerned to the person […] where all of the following apply’:

(i) ‘the treatment concerned is (I) immediately necessary for the protection of life of another person or persons, or (II) necessary for protection from an immediate and serious threat to the health of another person or persons;
(ii) the involuntarily admitted person requires the treatment concerned;
(iii) there is no alternative safe and effective treatment available;
(iv) it is likely that the condition of the involuntarily admitted person will benefit from such treatment’ (Section 51(1))

Finally, ‘where an application for a treatment order under *subsection (1)* is before the High Court, the Court may, pending its determination on the application, of its own motion or on the application of any person, give such interim directions as it sees fit as to the care and treatment of the person who is the subject of the application but any such direction shall cease to have effect immediately on the determination by the Court of the application’ (Section 51(4)).

## Issues raised by proposals for consent to treatment for involuntary patients

The arrangements outlined in the 2024 Bill would be a significant change from the 2001 Act. They are convoluted, inconsistent, and profoundly inadequate for the needs of people with serious mental illness who lack capacity and decline all forms of treatment. The Assisted Decision-Making (Capacity) Act 2015 does not permit any intervention with which a person does not cooperate, even if their non-cooperation occurs without capacity. The 2015 Act cannot meet needs that were previously met by the 2001 Act, as the 2024 Bill appears to imagine it can, to a significant extent.

Under the new proposals, even a Circuit Court treatment order could not be ‘contrary to the known will and preferences of the person concerned’ (Section 47(1)(c)). In such a case, a High Court application would be needed. However, relying on High Court orders for involuntary patients who lack capacity and decline to cooperate with care would lead to two parallel legal processes for such patients: mental health review boards reviewing detention orders and the High Court issuing treatment orders. This would be deeply ironic: doctors deciding who is detained and judges deciding who is treated. At worst, one might have imagined the opposite scenario, but certainly not this one.

Even if these proposals were feasible and coherent (which they are not), they would lead to impenetrable legal complexity, considerable delay, and substantial additional cost, given that there were 1951 involuntary admissions and 565 patients regraded from voluntary to involuntary in 2023 [7] (p. 64). Most of all, there would be inevitable inconsistency between the two legal processes for some involuntary patients who lack capacity and decline all forms of treatment: what if a mental health review board affirmed their detention, but the Court declined to issue a treatment order? Or vice versa?

The 2024 Bill adds yet more complexity and inconsistency by adding that, if an involuntary patient has been determined to lack capacity, ‘treatment may be administered […] for a period not exceeding 21 days following the making of an involuntary admission order […] where such treatment is (a) immediately necessary for the protection of life of the person or that of another person, or (b) necessary for protection from an immediate and serious threat to the health of the person, or that of another person, and there is no alternative safe and effective treatment available’ (Section 49(1)). The treatment ‘shall be reviewed at least once every 7 days, by the responsible consultant psychiatrist and the capacity of the person concerned shall be regularly reviewed by the responsible consultant psychiatrist or, on his or her direction, by another mental healthcare professional and in any event no longer than every 7 days’ (Section 49(4)). This appears inconsistent with Section 47(2) which does not place a 21-day limit on treatment while awaiting assessments of capacity or a Court hearing.

Finally, ‘where there is a valid advance healthcare directive in respect of a person in relation to the specific treatment proposed, the specific treatment shall not be administered if the advance healthcare directive specified that there is no consent to the treatment concerned or the designated healthcare representative duly authorised under an advance healthcare directive, refuses to consent to its administration’ (Section 49(6)).

Overall, these provisions for the treatment of involuntary patients require extensive revision to ensure that (a) consent is required from involuntary patients with capacity; (b) timely treatment and support are available to all who need them and (c) *mental health legislation* provides for treatment of involuntary patients with severe mental illness who lack capacity and do not cooperate with any form of treatment (and for whom the supports of the 2015 Act are, by definition, insufficient). Instead of requiring High Court applications for all such patients, *mental health legislation* should provide that treatment can be administered if it is immediately necessary.

In other words, the threshold for involuntary treatment should be the same as that for involuntary admission, i.e., the new Section 12(1)(b)*.* This should apply from the moment of the person’s arrival in the registered acute mental health centre and could be reviewed by mental health review boards, rather than requiring psychiatrists to apply to the High Court for all involuntary patients who lack capacity and do not cooperate with care.

This is a predictable and common clinical situation that the 2015 Act was not designed to address and it simply cannot do so, either legally or clinically. It is neither practical nor humane to require High Court hearings for all such patients. Mental health legislation should be designed to address this common scenario, with appropriate safeguards. The 2024 Bill is both inconsistent and enormously complex on this matter. These provisions require revision in the interests of coherence, common sense, patients’ rights, and access to care.

Moreover, the 2024 Bill’s provisions governing ‘treatment refusal’ are similarly impractical and convoluted (Section 50). They appear to suggest that, if an involuntary patient declines all treatment, detention ‘may’ be continued for ‘a period of 72 hours in order to build a therapeutic relationship’, but only if the patient has been detained on the basis of need for treatment (Section 12(1)(b)) rather than risk (Section 12(1)(a)). Again, this distinction is regrettable, inordinately convoluted and not evidence-based. This provision in the Bill makes no clinical sense and rests uneasily with the Bill’s other proposals about treatment for detained patients, which are similarly inconsistent, illogical and curiously divorced from clinical realities. Alternative proposals are outlined in this paper’s “Discussion” section (below).

## Restrictive practices

The 2024 Bill states that, ‘where a specified person applies a restrictive practice in respect of a voluntarily admitted person, the admission status of the voluntarily admitted person shall be reviewed pursuant to *section 37* [‘Power to detain voluntarily admitted person who fulfils criteria for involuntary admission’; above] as soon as possible after such application, but no later than 24 hours after the application concerned’ (Section 52(1)).

The Bill defines ‘seclusion’ as ‘the placing or leaving of a person in any room in which he or she is prevented from leaving freely or cannot otherwise leave freely’ (Section 2). A person can be placed in seclusion only if seclusion complies with relevant regulations (Section 53(d)) and:

- ‘The seclusion is ordered and initiated by a relevant health professional’ (Section 53(a));
- ‘The seclusion is applied in respect of the person by a relevant health professional or a specified person under the direct supervision of a relevant health professional’ (Section 53(b)); and
- ‘The application of such seclusion is determined by the relevant health professional, in accordance with regulations made under *section 57* [‘Regulations concerning restrictive practices’; below], to be necessary where there is an immediate threat of serious harm to the person or to another person, and such seclusion is, to prevent such a threat’ (Section 53(c)).

‘Mechanical restraint’ is ‘the application of any mechanical means of bodily restraint to a person in which a garment or a mechanical device restricts, prevents or otherwise limits a person’s freedom of movement or access to his or her own body’ (Section 2). Mechanical restraint can be applied only if it complies with relevant regulations (Section 54(d)) and:

- ‘The restraint is ordered and initiated by a consultant psychiatrist only’ (Section 54(a));
- ‘The restraint is applied in respect of the person by a relevant health professional or a specified person under the direct supervision of a relevant health professional’ (Section 54(b)); and
- ‘The application of such restraint is determined by the consultant psychiatrist, in accordance with regulations made under *section 57*, to be necessary where there is an immediate threat of serious harm to the person or to another person, or where it is necessary for the administering of treatment to the person concerned’ (Section 54(c)).

‘Physical restraint’ is ‘the application of any bodily restraint to a person where the intention is to restrict, prevent or otherwise limit a person’s freedom of movement or access to his or her own body’ (Section 2). Physical restraint can be applied only if it complies with relevant regulations (Section 55(d)) and:

- ‘The restraint is ordered and initiated by a relevant health professional’ (Section 55(a));
- ‘The restraint is applied to the person by a relevant health professional or a specified person under the direct supervision of a relevant health professional’ (Section 55(b)); and
- ‘The application of such restraint is determined by the relevant health professional, in accordance with regulations made under *section 57*, to be necessary where there is an immediate threat of serious harm to the person or to another person, or where it is necessary for the administering of treatment to the person concerned’ (Section 55(c)).^5^

The inclusion of treatment as a reason for physical restraint under specific circumstances is a welcome correction to the definition of physical restraint presented by the Mental Health Commission in its 2022 *Code of Practice on the Use of Physical Restraint*, which defined physical restraint as:

The use of physical force (by one or more persons) for the purpose of preventing the free movement of a person’s body when the person poses an immediate threat of serious harm to self or others [8] (p.7).

It is worth noting that that definition does not appear in the 2001 Act. It does not specify treatment as a reason for physical restraint, although, as the Commission points out, ‘the 2001 Act does not impose a legal duty on staff working in mental health services to comply with Codes of Practice’ (p. 1). In addition, the Code *does* list ‘treatment’ as a reason for physical restraint in its Appendix 2.

Against this background, it is welcome that the 2024 Bill seeks to clarify this matter in primary legislation by articulating treatment as a reason for physical restraint in specific, limited circumstances. This matter was not addressed in the 2001 Act, so the position of the 2024 Bill on this point is the *only* indicator of the intent of the legislators in this area. As such, the position of the 2024 Bill on this matter could usefully inform current regulatory and legal proceedings to a significant degree.

The 2024 Bill goes on to specify that ‘a restrictive practice may only be applied in respect of a person (a) in rare and exceptional circumstances, (b) where there is no safe alternative for the person, (c) where the application is proportionate to the immediate threat of serious harm to the person or to another person that has been assessed by the person who orders and initiates the practice to exist, and (d) for the shortest duration possible’ (Section 56(1)). Staff must keep appropriate records (Section 56(3)) and explain the reasons for, expected duration of and circumstances that would lead to discontinuation of the restrictive practice ‘to the person in a manner in which that person is reasonably expected to understand’ (Section 56(2)).

Section 185 states that ‘where a person has been admitted to a registered acute mental health centre, the person may nominate a person (in this section referred to as a “nominee”) on his or her behalf to be provided with information in relation to his or her treatment under this Act or on the application of a restrictive practice’ (Section185(1)). ‘Regarding the application of a restrictive practice, the views of (a) the person, and (b) where appropriate, a nominee nominated under *subsection (1)*, shall be taken into account by the consultant psychiatrist or the relevant health professional responsible for ordering the application of a restrictive practice and those views shall be recorded in writing in his or her medical record and care plan’ (Section 185(6)).

Finally, the Mental Health Commission ‘may, with the consent of the Minister, make regulations providing for the application of restrictive practices’ (Section 57(1)), with particular emphasis on ‘dignity and respect’ (Section 57(2)(a)), ‘welfare and dignity […] including the person’s right to privacy, bodily integrity and autonomy and insofar as possible, respect for the person’s will and preferences’ (Section 57(2)(c)), relevant ‘procedures’ (Section 57(2)(d)), ‘records’ (Section 57(2)(e)), ‘monitoring’ (Section 57(2)(f)), ‘renewal’ (Section 57(2)(g)), ‘discontinuation’ (Section 57(2)(h)), ‘facilities and staff’ (Section 57(2)(i)), ‘the training and experience of relevant health professionals or specified persons’ (Section 57(2)(j)), ‘clinical governance of a restrictive practice, including written policies’ (Section 57(2)(k)), ‘use of CCTV or other electronic monitoring’ (Section 57(2)(l)),^6^ ‘communication with any nominated person regarding the application of a restrictive practice, including how the views of such persons or the person upon whom the practice is administered are considered’ (Section 57(2)(m)), and ‘any other matters which are necessary or expedient’ (Section 57(2)(n)).

It will be crucial that the term ‘immediate’ is defined in secondary legislation to ensure that patients who lack capacity and are resisting treatment but whose condition may deteriorate over the course of days rather than hours will get timely treatment. No patient should become so unwell that they are placed at risk of additional complications and increased length of stay while waiting for the risk to themselves or others to become immediate. Clarification would aid consistent application of the regulation of restrictive practice nationally, help protect rights and support clinicians in providing ethically informed care.

## Mental Health Commission and regulation of mental health services

Part 5 of the 2024 Bill deals with the Mental Health Commission and addresses, *inter alia*, the ‘continuance in being’ of the Commission (Section 92) (see “Discussion” section of this paper, below) and ‘functions of Commission’ (Section 93).^7^ Chapter 6 of Part 5 concerns the ‘Inspector, inspections and inquiries’ and states that the ‘Chief Inspector’ shall be ‘a consultant psychiatrist’ (Section 127(2)). The ‘Functions of Chief Inspector’ (Section 128) are outlined in Box 3.

**Box 3:** Functions of the Chief Inspector of Mental Health Services under Ireland’s Mental Health Bill 2024 (Section 128)

‘The functions of the Chief Inspector shall be—
(a) to visit and inspect
(i) every registered acute mental health centre at least once in each registration period of 3 years,
(ii) every registered community mental health centre at least once in each registration period of 5 years, and
(iii) every registered community mental health service at least once in each registration period of 5 years,
(b) to prepare and publish inspection reports in accordance with *section 133* [‘Inspection reports’],
(c) to visit and inspect every mental health service in respect of which an application for registration, other than a registration referred to in *paragraph (d)*, has been received by the Commission and to produce a report regarding its suitability for registration,
(d) to visit a registered mental health service that has applied to renew its registration, in accordance with *section 149 (5)* [‘Renewal of registration’],
(e) to carry out an inquiry in accordance with *section 135* [‘Inquiries’];
(f) to ensure compliance in accordance with *Part 6* [‘Regulation of Mental Health Services’], and
(g) to carry out an annual review of mental health services and prepare and submit a report to the Commission in accordance with *section 134* [‘Annual review and report of Chief Inspector’].

Other relevant provisions in the 2024 Bill govern the ‘Acting Chief Inspector’ (Section 129), ‘Assistant Inspectors of Mental Health Services’ (Section 130), ‘powers of inspectors’ (Section 131), ‘duties of inspector when making an inspection’ (Section 132), ‘Inspection reports’ (Section 133), ‘annual review and report of Chief Inspector’ (Section 134), inquiries (Section 135), ‘penalty for obstruction of Inspector’ (Section 136) and ‘legal privilege’ (Section 137)

Part 6 of the Bill concerns ‘Regulation of Mental Health Services’ and provides for registers of ‘acute mental health centres’ (Sections 139, 142, and 146), ‘community mental health centres’ (Sections 140, 143, and 147) and ‘community mental health services’ (Sections 141, 144 and 148), among other matters.^8^

Chapter 3 of Part 6 governs the ‘Registered Proprietor, Registered Person, Responsible Person and Clinical director’, including provisions relating to the ‘registered proprietor’ (Section 161), ‘delegation of functions of registered proprietor’ (Section 162), a ‘registered person’ (Section 163), the ‘clinical director’ (a ‘consultant psychiatrist’) (Section 165(1)) and a ‘responsible person’ who ‘shall have the following responsibilities namely: (a) carrying on the day-to-day operation of the registered mental health service; (b) reporting to the registered proprietor and to the Commission on the day-to-day operations of the registered mental health service as necessary and when requested to do so, including in relation to any compliance with this Act; (c) ensuring compliance by members of the staff of the registered mental health service with this Act and any regulations made thereunder and any conditions imposed upon the registration of the service; (d) liaising with the Commission from time to time and when requested to do so by the Commission’ (Section 164(3)).

Chapter 4 of Part 6 concerns ‘compliance notices’ (Section 166); Chapter 5 addresses ‘Closure, Cancellation and Taking Charge’ (Sections 167 to 174) and Chapter 6 presents provisions governing ‘regulations for registration, operation and management of mental health services’, stating that the Minister for Health may ‘make regulations in relation to the registration, operation and management of a registered mental health service including matters relating to the care and treatment of persons in that service’ (Section 175(1)).

The Bill’s ‘miscellaneous’ provisions address ‘codes of practice’ about many matters, including the application of the Assisted Decision-Making (Capacity) Act 2015 to involuntary patients (Section 177(1)(a))^9^; ‘use of electronic signatures’ (Section 178) and ‘care plans for registered acute mental health centres’ (Section 179) (Box 4). A ‘care plan’, ‘in relation to an adult’ is ‘a plan prepared under *section 179* by a member of a person’s multidisciplinary team, and where possible, in consultation with the person the subject of the plan’ (Section 2).

**Box 4:** Care Plans for Registered Acute Mental Health Centres under Ireland’s Mental Health Bill 2024 (Section 179)

(1) ‘A member of a person’s multidisciplinary team shall, following a comprehensive assessment of a person in a registered acute mental health centre, prepare a care plan based on that assessment no later than 14 days after that person’s admission to the centre.
(2) Subject to *subsection (3)*, a care plan prepared under *subsection (1)* shall—
(a) identify the person’s care and treatment needs required to meet any recovery goals identified,
(b) outline appropriate goals towards the effective recovery of the person,
(c) specify the resources required to achieve the care and treatment identified in the plan, and
(d) have due regard to the will and preferences of the person, the subject of that care plan.
(3) A member of a person’s multidisciplinary team shall—
(a) review the care plan on a regular basis, with the frequency of review based on the individual needs of the person concerned, and
(b) where necessary or relevant, revise the plan after consultation and, insofar as possible, in consultation with the person concerned and other members of the multidisciplinary team as appropriate.
(4) A member of a person’s multidisciplinary team may, where the consent of the person concerned has been given, consult with—
(a) the nominated person (if any) of the person concerned, or
(b) any other person, where appropriate.
(5) A care plan prepared under *subsection (1)* or revised under *subsection (3)(b)* shall be—
(a) signed where possible or appropriate by the person the subject of the plan,
(b) retained with the person’s medical records for such period as may be specified by the Commission, and
(c) accessible to the person the subject of the plan.’

The 2024 Bill adds that ‘the Minister [for Health] may make regulations concerning care plans which may provide for’ a broad range of matters, including ‘the content of a care plan, including the setting of goals’ (Section 181(a)), ‘the resources required to achieve any goals’ (Section 181(b)), ‘the review of a care plan by a multidisciplinary team’ (Section 181(c)) and various other matters (Sections 181(d) to (i)). These provisions are further considered in the “Discussion” section of this paper (below).

Finally, additional provisions in the 2024 Bill govern ‘data protection’ (Chapter 2; Section 182); ‘processing of personal data and special categories of personal data’ (Section 183); ‘regulations concerning data protection’ (Section 184); ‘records to be maintained for registered mental health services’ (Section 187) and ‘registered acute mental health centres’ (Section 188); ‘offences and penalties’ (Section 189); ‘time limit where offence may be prosecuted in summary proceedings only’ (Section 190); ‘liability for offences by body corporate’ (Section 191); ‘offence of false or misleading information’ (Section 192); fees (Section 193); matters relating to ‘legal aid’ (Chapter 5)^10^ and relevant amendments to the Non-Fatal Offences against the Person Act 1997 (Section 201) and Assisted Decision-Making (Capacity) Act 2015 (Section 202).

## Discussion

Ireland’s Mental Health Bill 2024 proposes several useful revisions to Irish mental health legislation including certain new definitions (e.g. ‘mental disorder’, ‘treatment’) (Section 2) and various provisions governing specific practices (e.g. ‘physical restraint’) (Section 55). Further revision is, however, needed. Certain suggestions were already provided throughout this paper. This discussion focuses on four key areas which merit particular attention in the preparation of the final Act: (a) proposed criteria for involuntary admission (Section 12), (b) proposed provisions for treatment of involuntary patients (Chapter 3 of Part 3), (c) proposed provisions governing particular formats of care plans (Sections 179 and 181) and (d) the proposal that the Mental Health Commission should continue to exist as a stand-alone entity (Section 92).

- Proposed criteria for involuntary admission

The 2024 Bill proposes two separate criteria for involuntary admission based on the presence of ‘mental disorder’ and either additional risk criteria (Section 12(1)(a)) or additional treatment criteria (Section 12(1)(b)) (Box 2). This requires revision.

The remarkable persistence of risk criteria in mental health legislation reflects a long-standing trend whereby legislators and society more broadly seek to use psychiatry as a socially convenient repository of ill-defined risk that is not clearly linked with treatable mental illness [9]. This is reflected in mental health legislation around the world, including in Ireland, despite the fact that so-called risk assessment lacks a firm evidence base [10]. Nobody can predict the future with sufficient reliability to justify deprivation of liberty, even in so-called high-risk populations [3].

In terms of the proposed criteria for involuntary admission in the 2024 Bill, the treatment criteria are clear, pragmatic and focused on treatment of illness—a task for which psychiatry has an evidence base that is at least as strong as that in other areas of medicine and often stronger [1, 2, 4]. The proposed risk criteria, by contrast, are conceptually unsound and rely on assessment of risk of future harm—a task which lacks a firm evidence base. In the final Act, the proposed treatment criteria (Section 12(1)(b)) should be retained and the proposed risk criteria (Section 12(1)(a)) should be deleted, so that legislation focuses on treatment of mental illness (which psychiatry can do) rather than assessment of risk of future harm (which it cannot) [11].

(b) Treatment of involuntary adult patients under mental health legislation

The 2024 Bill requires considerable revision in relation to the treatment of involuntary patients who lack capacity and decline care. In essence, it should be possible to provide treatment at all times after the person’s arrival at the ‘registered acute mental health centre’. That is the purpose of psychiatric admission and the essential purpose of all psychiatry: treatment of mental illness. Legislation should facilitate accountable, person-centred care, rather than obstruct or delay it. Extensive problems with the relevant proposals in the 2024 Bill were already outlined above, so this discussion will present an alternative paradigm for consideration.

Immediately after a person arrives at a registered acute mental health centre with an Application and Recommendation already completed, and while awaiting completion of an Admission Order by the consultant psychiatrist (a period which cannot exceed 24 hours), the following should apply (similar to the suggestions of the 2015 Expert Group):

- If the person possesses decision-making capacity for treatment decisions, treatment should occur only with their consent.
- If the person appears to lack decision-making capacity for treatment decisions (following such assessment of decision-making capacity as is possible in these circumstances, and following the provision of such decision-making supports as are possible in these circumstances), treatment can be provided without consent if such treatment is ‘(a) immediately necessary for the protection of life of the person or that of another person, or (b) necessary for protection from an immediate and serious threat to the health of the person, or that of another person’ (in the wording of Section 49(1) of the 2024 Bill).

After the Admission Order is completed by the consultant psychiatrist, the following should apply:

- If the person possesses decision-making capacity for treatment decisions, treatment should occur only with their consent.
- If the person appears to lack decision-making capacity for treatment decisions, their decision-making capacity is to be assessed by the consultant psychiatrist immediately after completing the Admission Order. If, following the provision of such decision-making supports as are possible in the circumstances, the person still lacks decision-making capacity for treatment decisions, treatment can be provided without consent based on the same criteria as the treatment criteria for involuntary admission (i.e. that ‘the person has a mental disorder, the nature and degree of which is such that (i) he or she requires care and treatment immediately,^11^ (ii) the care and treatment required to be given to the person cannot be given to that person other than in a registered acute mental health centre and (iii) the reception, detention and care and treatment of the person concerned in a registered acute mental health centre would be likely to benefit the condition of that person’) (Section 12(1)(b)).
- In addition: (a) the involuntary patient should be advised about the supports of the Assisted Decision-Making (Capacity) Act 2015 of which they can avail if they wish, with the assistance of their legal advisor (because such supports might help them to regain decision-making capacity faster); (b) the involuntary patient’s decision-making capacity should be re-assessed at least every 7 days for the first 2 months by the responsible consultant psychiatrist (in a multi-disciplinary context), and monthly thereafter, for as long as they remain an involuntary patient and lack decision-making capacity; (c) the independent consultant psychiatrist engaged by the Mental Health Commission ahead of the Mental Health Review Board hearing (i.e. within 21 days) should assess the patient’s decision-making capacity as part of their report and, if their assessment differs from that of the responsible consultant psychiatrist, they should inform the responsible consultant psychiatrist immediately, with appropriate action taken; and (d) the issue of decision-making capacity should be reviewed by a Mental Health Review Board, along with other relevant matters, at their scheduled hearing, within 21 days.

This proposed scheme would protect patients’ rights in a more comprehensive way (including rights to liberty, bodily integrity and treatment); ensure patients are aware of the supports of the 2015 Act (if needed); use more fully the existing resource of their legal advisors (who can help patients to access the supports of the 2015 Act); ensure that decision-making capacity is reviewed regularly during involuntary admission (in a multi-disciplinary context); use more fully the existing resource of independent consultant psychiatrists engaged by the Mental Health Commission ahead of Mental Health Review Board hearings (by acting on their decision-making capacity assessments immediately); use more fully the existing resource of Mental Health Review Boards (by adding appropriate oversight in relation to decision-making capacity) and remove the 2024 Bill’s requirement for routine High Court hearings for involuntary patients who lack decision-making capacity and decline all treatment (which is a common clinical scenario).

Most of all, this paradigm would facilitate prompt, person-centred treatment and the alleviation of suffering, with robust protection of rights. Involuntary patients would, of course, still have free legal aid, routine Mental Health Review Board hearings and access to the Circuit Court and High Court, as outlined in the Bill.

(c) Care plans in the Mental Health Bill 2024

The position of care plans in the 2024 Bill requires significant reconsideration. While the *Report of the Expert Group on the Review of the Mental Health Act 2001* placed a certain emphasis on care plans [6] (pp. 65–67), that report reflected ‘the majority views of the Expert Group members on each issue considered’, rather than unanimity (p. 86).^12^ Even in the context of those recommendations, however, the position accorded to a particular kind of care plan in the 2024 Bill should be reconsidered and revised or removed.

As they presently stand, the relevant provisions in the 2024 Bill (outlined above) represent significant regulatory overreach into individual clinical care, lack an evidence base, have insufficient regard for pre-existing or alternative models of care and engagement, elevate Procrustean standardisation over efficacy,^13^ disregard the wishes of service-users who do not agree with such care plans and constitute an unprecedented intrusion by the State into individual care, treatment and support. In addition, the Bill accords extraordinary power to the Minister for Health, permitting them to regulate ‘the *content* of a care plan’ (Section 181(a)) (emphasis added); the content of treatment is usually a clinical matter that is negotiated between the patient and their clinicians, rather than determined by politicians or regulators. The 2024 Bill seeks to change that.

These provisions also demonstrate a curious lack of awareness of the content and nature of existing clinical records and existing models of care planning. The requirement to *duplicate* material from medical records into care plans is made explicit in the Bill (e.g. Section 185(6)). In resource-stretched mental health services, duplicating information from clinical records into a specific kind of care plan with a particular prescribed format (as opposed to care plans which were previously used or simply using clinical records themselves as care plans) already commands significant opportunity cost. Such duplication diverts scarce human resources away from therapeutic activities and patient engagement and towards meeting regulatory requirements which lack systematic evidence of benefit.

The new provisions governing care plans, if carried through, would greatly magnify that problem and further diminish patient care. If these provisions remain unrevised in the final Act, it is possible that services will re-title existing clinical records as ‘care plans’ (to avoid duplication) and make whatever modifications are necessary to their format solely in order to meet the escalating regulatory requirements. This might minimise the negative impact of these regulations on services. The better solution would be to entirely remove these provisions from primary legislation, which is ill-suited to micro-managing the subtleties of individual care at this level of detail.

Every patient should be entitled to a plan of care which is shaped by systematic evidence of benefit (rather than ideology); the applied expertise of patients, clinicians and families co-working together (rather than routine interventions by judges, regulators, legislators or politicians at the level of individual treatment) and leveraging existing models of care planning and support (rather than imposing highly particular, arbitrary formats of care plans). This cannot be achieved by foisting Procrustean, non-evidence-based requirements on services as legal obligations in primary legislation or having the Minister for Health regulate the content of individual clinical care.

(d) The continuance of the Mental Health Commission

Section 92 of the 2024 Bill provides for the ‘continuance in being’ of the Mental Health Commission. This provision merits reconsideration before the Act is finalised. The Mental Health Commission was created by the Mental Health Act 2001 and was arguably necessary at that time in order to initiate the radical innovations of the 2001 Act, especially the introduction of mental health tribunals. These measures are now up and running, and the 2024 Bill proposes incremental changes, rather than radical shifts, in these tasks. The same applies to inspections, which will be expanded under the 2024 Bill but are not a new concept. Against this background, and for various other reasons, the continuance in being of the Mental Health Commission as a stand-alone entity merits reconsideration.

It is increasingly recognised that mental health is intimately bound up with physical health, and that mental health services should be more closely linked with general health services in order to achieve better outcomes. From a clinical and biological perspective, it is not possible to separate mental health from physical health, so the persistence of a Cartesian dualism in regulation does not reflect lived reality. With this in mind, it would be sensible and helpful if all parts of the health system were subject to the same regulatory body, albeit with appropriate adjustments to suit each area. This is already the case across the diverse areas of health and social care which are regulated by the Health Information and Quality Authority (HIQA) according to the regulatory needs of each.

Against this background, it is not at all clear that the Mental Health Commission should continue to exist as a stand-alone body. In fact, it would make greater sense for its functions and expertise to be transferred to HIQA. Such a shift would help to standardise regulatory functions across all of health and social care, reduce the separateness and stigma that are still associated with mental health services and likely deliver significant economies of scale. Most importantly, it would facilitate greater mutual learning, pooling of expertise and systematic engagement with the evidence-base for regulation as part of a larger organisation, for the benefit of all. There would also be helpful synergies and enhanced coordination across the system as a whole, as the accumulated expertise of the Mental Health Commission would undoubtedly enhance HIQA more generally, and vice versa.

Relocating the Decision Support Service (DSS) to HIQA would also likely deliver benefit. The DSS was created under the Assisted Decision-Making (Capacity) Act 2015 and is currently situated in the Mental Health Commission. Relocating the DSS to HIQA as part of this shift would deliver many similar benefits and would also better reflect the remit of the DSS and its stakeholders. Mental illness is by no means invariably associated with lack of decision-making capacity, and many people who lack decision-making capacity do not have a mental illness [12]. As a result, locating the DSS in HIQA (rather than in the Mental Health Commission) would more accurately reflect the broad remit of the DSS across all elements of health and social care, in addition to other benefits.

## Conclusions

The Mental Health Bill 2024 proposes several positive changes to the Irish mental health legislation, but further revision is needed to better provide care and better protect rights. Key suggestions for revisions to the final Act, which were detailed earlier in this paper, include:

- Retaining the treatment criteria in the Bill’s proposed criteria for involuntary admission (Section 12(1)(b)), but deleting the risk criteria (Section 12(1)(a)), so that legislation focuses on treatment of mental illness (which psychiatry can do) rather than assessment of risk of future harm (which it cannot)
- Revising proposed provisions for the treatment of involuntary patients (Chapter 3 of Part 3) to ensure that *mental health legislation* provides for timely, accountable treatment and support for all, including involuntary patients with severe mental illness who lack decision-making capacity and decline treatment (see above for specific suggestions)
- Omitting detailed provisions about the content of individual treatment plans from primary legislation (Sections 179 and 181), which is ill-suited to micro-managing the subtleties of individual care; in addition, individual clinical care should be evidence-based and free from political interference
- The Mental Health Commission (which was created by the Mental Health Act 2001) and the Decision Support Service (which was created by the Assisted Decision-Making (Capacity) Act 2015) should be incorporated into the Health Information and Quality Authority

These proposed revisions to the Bill would help provide care and protect rights for everyone on an equal basis, with appropriate recognition of each individual’s unique needs for treatment and support. One size does not fit all.

These matters are especially important for involuntary patients who lack decision-making capacity and decline treatment, and for whom the Assisted Decision-Making (Capacity) Act 2015 does not (and was not designed to) provide comprehensive solutions. Mental health legislation can be aligned with the provisions and principles of the 2015 Act without relying on the 2015 Act to solve problems it was not designed to solve.

Relying on a convoluted, self-contradictory combination of the 2015 Act, Circuit Court and High Court for involuntary patients who lack decision-making capacity and decline treatment would be legally impossible, clinically impracticable and a *de facto* denial of the rights of people with serious mental illness and their families.

## Footnotes

1 Provisions regarding children are not outlined in this paper; these would be best examined by an author with greater clinical and legal expertise in that field.

2 ‘Mental healthcare professional’ is defined in Section 2.

3 The Mental Health Commission ‘shall prepare and publish a code of practice in relation to capacity assessments’ (Section 45(9)).

4 Section 48 governs electro-convulsive therapy (ECT) and states that the Mental Health Commission ‘shall, with the consent of the Minister, make regulations providing for the use of electro-convulsive therapy’ (Section 48(3)). The legislation does not explain why it chooses to treat ECT in a different way to other treatments and therefore perpetuate the stigma surrounding this evidence-based therapy [3].

5 The term ‘immediate threat of serious harm’ would benefit from greater definition. For example, does it apply in instances where the ‘threat’ is ‘immediate’, but the ‘serious harm’ is less so, e.g. a severely depressed involuntary patient who is not eating or drinking, and for whom electro-convulsive therapy is clinically indicated, and without which they will inevitably deteriorate and possibly die? In that instance, the threat is immediate even if the serious harm is not, but the serious harm is nevertheless proximal, substantial and preventable.

6 In such regulations, consideration should be given to balancing the benefits and risks of direct, one-to-one observation as opposed to remote monitoring, focusing on what is best for the patient.

7 The 2024 Bill outlines provisions governing functions of the Board of the Commission (Section 94), ‘membership of Board’ (Section 95), ‘casual vacancies’ (Section 96), ‘removal of member of Board’ (Section 97), ‘removal of all members of Board from office’ (Section 98), ‘meetings and procedures of Board’ (Section 99), ‘committees of Board’ (Section 100), ‘remuneration and expenses of members of Board and committees’ (Section 101), ‘membership of either House of Oireachtas, European Parliament or local authority’ (Section 102), the ‘Chief Executive Officer’ (Sections 103–108), staff of the Commission (Sections 109–113), ‘specialist advisers’ (Section 114) and ‘funding and accountability’ (Sections 115–126).

8 Additional provisions govern ‘carrying on of more than one registered mental health service’ (Section 145), ‘renewal of registration’ (Section 149), ‘representations’ (Section 150), ‘duration of registration’ (Section 151), ‘variation or addition of conditions’ (Section 152), ‘application by registered mental health service to vary or remove condition’ (Section 153), ‘removal of conditions by Commission’ (Section 154), ‘suspension and revocation of registration’ (Section 155), ‘appeals’ (Section 156), ‘duty to display certification’ (Section 157), ‘material amendment of registration’ (Section 158), ‘duty to notify change in information’ (Section 159) and ‘requests by Commission for information’ (Section 160).

9 Such codes ‘shall be admissible in evidence in any proceedings under this Act or before a court or tribunal’ (Section 177(9)); mental health review boards are not mentioned.

10 These include various interpretative matters (Section 194), ‘application of Chapter’ (Section 195), ‘panel of legal representatives and provision of legal aid’ (Section 196), ‘criteria for granting legal aid for appeal’ (Section 197), the ‘relationship between legal representative and person in receipt of legal aid’ (Section 198), that the ‘Commission may request certain information in respect of legal representation’ (Section 199) and ‘regulations concerning legal aid’ (Section 200).

11 It might be more accurate and useful to change immediately to ‘urgently’ in this section.

12 BDK was a member of the Expert Group on the Review of the Mental Health Act 2001.

13 Procrustes was a figure in Greek mythology who stretched people or shortened their legs so that they were all the same size; the term ‘Procrustean’ now refers to standardisation which has insufficient regard for the merits of pre-existing approaches, does not recognise the advantages of diverse or alternative models, is insufficiently responsive to changing contexts and/or ignores the negative effects of its particular (generally arbitrarily chosen) model of standardisation.
